# Correction: Roles of water in the formation and preparation of graphene oxide

**DOI:** 10.1039/d1ra90117f

**Published:** 2021-05-20

**Authors:** Qiang Zhang, Yuying Yang, Huiqing Fan, Liu Feng, Guangwu Wen, Lu-Chang Qin

**Affiliations:** College of Chemistry and Chemical Engineering, Shandong University of Technology China; College of Materials Science and Engineering, Shandong University of Technology China; Department of Physics and Astronomy, University of North Carolina at Chapel Hill USA

## Abstract

Correction for ‘Roles of water in the formation and preparation of graphene oxide’ by Qiang Zhang *et al.*, *RSC Adv.*, 2021, **11**, 15808–15816, DOI: 10.1039/D0RA10026A.

The authors regret that there was an error in the author affiliations in the original article. The correct affiliations are given here.

The Royal Society of Chemistry apologises for these errors and any consequent inconvenience to authors and readers.

## Supplementary Material

